# Impacts of the properties heterogeneity on 3D magnetic dusty nanofluids flow in porous enclosures with cylinders

**DOI:** 10.1038/s41598-022-13196-z

**Published:** 2022-06-01

**Authors:** Z. Z. Rashed

**Affiliations:** Mathematics Department, Faculty of Science and Arts, Jouf University, Qurayyat, Saudi Arabia

**Keywords:** Energy science and technology, Engineering, Mathematics and computing

## Abstract

This paper examines the controlling of the three dimensional dusty nanofluid flow using the two circular cylinders having different thermal conditions. The cylinders are located in the middle area while the location of the right cylinder is changeable. The 3D (three dimensional) cubic flow domain is filled by a non-Darcy porous medium and a magnetic field in Z-direction is taken place. The non-homogeneous two phase model of the nanofluid is applied while the permeability and thermal conductivity of the porous medium are assumed heterogonous. The current situation is represented by two systems of the equations for the nanofluid and dusty phases. The solutions methodology is depending on the 3D SIMPLE scheme together with the finite volume method. Here, It is focused on the distance between the cylinders $\delta\, (0.3\le \delta \le 0.6)$, the Darcy number $Da\, ({10}^{-2}\le Da\le {10}^{-5})$, the dusty parameter ${\alpha }_{d}\,(0.001\le {\alpha }_{d}\le 0.1)$, the average nano-parameter ${\phi }_{av}\,(0.01\le {\phi }_{av}\le 0.03)$. The major outcomes indicating to that the flow can be well controlled using the inner isothermal cylinders. Also, the cases of the heterogeneity in $X{-}Y$ and $X{-}Z$ directions give the lowest values of ${Nu}_{av}$. Both the flow and heat transfer rate are enhanced as $\delta$ is increased.

## Introduction

Examining of the natural convection flow within closed domains in porous media has received much attention from many researchers because of its widespread applications in various fields, such as engineering systems, electronics cooling, geothermal reservoirs, nuclear reactors, regenerative heat exchangers, electric machinery, solar collectors, air conditioning, and chemical industries. Such applications have been found in several studies, for example in^1–5^. On the other hand, nanofluids have gained great importance over the past years due to superior performance on the improvement of thermal conductivity compared to basic fluids. The nanofluid is defined as a fluid consists of a basic liquid mixture with solid nanoparticles (nanosized solid particles such as Cu, Ag, CuO and Al_2_O_3_) to the base fluid of dimensions less than 100 nm. There are several published papers on dealt with thermophysical properties of nanoparticles, development and preparation can be found in^6–11^. Afterward, different numerically and experimentally studies for different surfaces and media were presented; for example but not limited to^12–19^.

May by reviewing the existing literatures on the natural convection of nanofluids, it was found that few researchers have studied the nanoparticles in a 3D domains with/without porous enclosure. Jelodari, and Nikseresht^20^ discussed the effect of the Lorentz force on the thermal performance of nanofluids in a cubic cavity. They used a numerical technique based on the finite volume method. They observed that the conduction heat transfer is predominated when the concentration of the nanoparticles is increased up to 6%. Sajjadi et al.^21^ investigated MHD natural convection in a cubic cavity with sinusoidal boundary conditions. They indicated to with the increase of the Rayleigh number and nanoparticles’ volumetric fraction, the Nu is increased. Wang et al.^22^ analyzed the natural convection of the nanofluids in a partially heated cubic enclosure. They found that if the aspect ratio increases, the average Nusselt number, and the heat transfer are decreased. Sheikholeslami et al.^23^ discussed the lattice Boltzmann method for the roles of magnetic field on the free convection of the nanofluids in the porous media using the non-Darcy model. From the results, it is seen that the increased Darcy number leads to the temperature boundary layer thickness becomes thinner. Sheremet and Pop^24^ investigated the natural convection in a heated cubical cavity under the Marangoni effect. They applied the finite difference method then discussed the impacts of the controlling parameters on the velocity, temperature, nanoparticles volume fraction and the average Nusselt number. Sheikholeslami et al.^25^ considered a cubical enclosure in the existence of a magnetic field with hot sphere obstacle in the flow domain. They found that the increase of Darcy number leads to the thermal boundary layer becomes thicker. Alsabery et al.^26^ discussed the unsteady 3D natural convection heat transfer inside a wavy porous cubical area using a Galerkin weighted residual scheme based on the finite element method. They observed that the increasing in Da causes that the Nusselt number is significantly increased.

On the other side, many researchers concentrated on the case of containing small solid particles like dust particles. This mixture type called dusty fluid. Study the properties of these types of liquids have wide range of applications such that, cooling systems, flows in rocket tubes, environmental pollutants and in engineering and sciences…etc. These applications can be found in Marble^27^ and Rudinger^28^. Afterward several studies have been extended to the case of the dusty nanofluids. Naramgari and Sulochana^29^ studied the dusty nanofluid flow over a stretching surface with effect of the magnetic force. Their governing system is solved numerically by using Runge-Kutta based shooting technique. They found that an increase in Hartmann number help to decrease the friction factor. Begum et al.^30^ studied the problem of bioconvection boundary layer flow of two-phase dusty nanofluids. They used numerical solutions and found that the rising in the buoyancy ratio and mass concentration parameters leads to a reduction in the skin friction coefficient. Siddiqa et al.^31^ investigated the natural convection flow of two phase dusty nanofluid along a vertical wavy surface. From the results, the temperature profiles tend to increase with the growing in modified diffusivity ratio. Gireesha et al.^32^ discussed the Hall current effect on the dusty nanofluid flow and used the Runge–Kutta–Fehlberg method coupled with shooting algorithm. Their results pointed to that the Hall effects lead to accelerate the velocities.

Mishra et al.^33^ investigated the magnetic field effect on the dusty nanofluids in a porous medium. They interested in the influences of the governing parameters on the skin friction coefficient and the heat transfer rate for both the fluid and dusty phases. Rashid et al.^34^ studied the mixed convection in a porous medium with radiation effect. They concluded that an increase of the volume fraction of the nanoparticles leads to an enhancement in the Nusselt coefficient. Recently, Rashed and Ahmed^35^ discussed the peristaltic flow of a dusty nanofluid in curved channels.

Non-homogeneous model has been used by few researchers to study natural convection of the nanofluids saturated porous cubic cavities. Zhuang and Zhu^36,37^ studied the buoyancy Marangoni convection of non-Newtonian nanofluids with a heterogeneous porous medium, numerically, by applying the finite volume method. They found that the level of heterogeneity controls the entropy generation and decreases the heat transfer rate. Rashed et al.^38^ investigated the unsteady 3D nanofluid flow within a cubic enclosure filled with a heterogeneous porous medium and they discussed different cases. Other related works for the current study are the investigations presented in^39–51^.

As evident from the above literature review, no research yet dealing with the unsteady natural convection flow of dusty nanofluid flow within cubic enclosure in a porous medium with hot and cold cylinders. Hence, the aim of this study is investigating the dusty nanofluid flow within the cubic enclosure in a porous medium with hot and cold cylinders in the presence of the magnetic field and a heterogeneous porous medium influences. During this simulation, we discuss the effects different parameters of problem on the temperature, velocity for dusty/nanofluid phases and the average Nusselt number. The novelty of the current study is appearing in collecting important aspects such as 3D dusty nanofluids flow using the non-homogeneous nanofluids model together with the case of the heterogeneous porous medium. Further, this study can be related to important practical applications such as thermal insulation, filtration processes, geothermal systems, oceanography, building insulation, geothermal reservoirs, geophysics, separation processes in chemical industries and electronic equipment cooling.

## Governing equations

**Figure 1:**
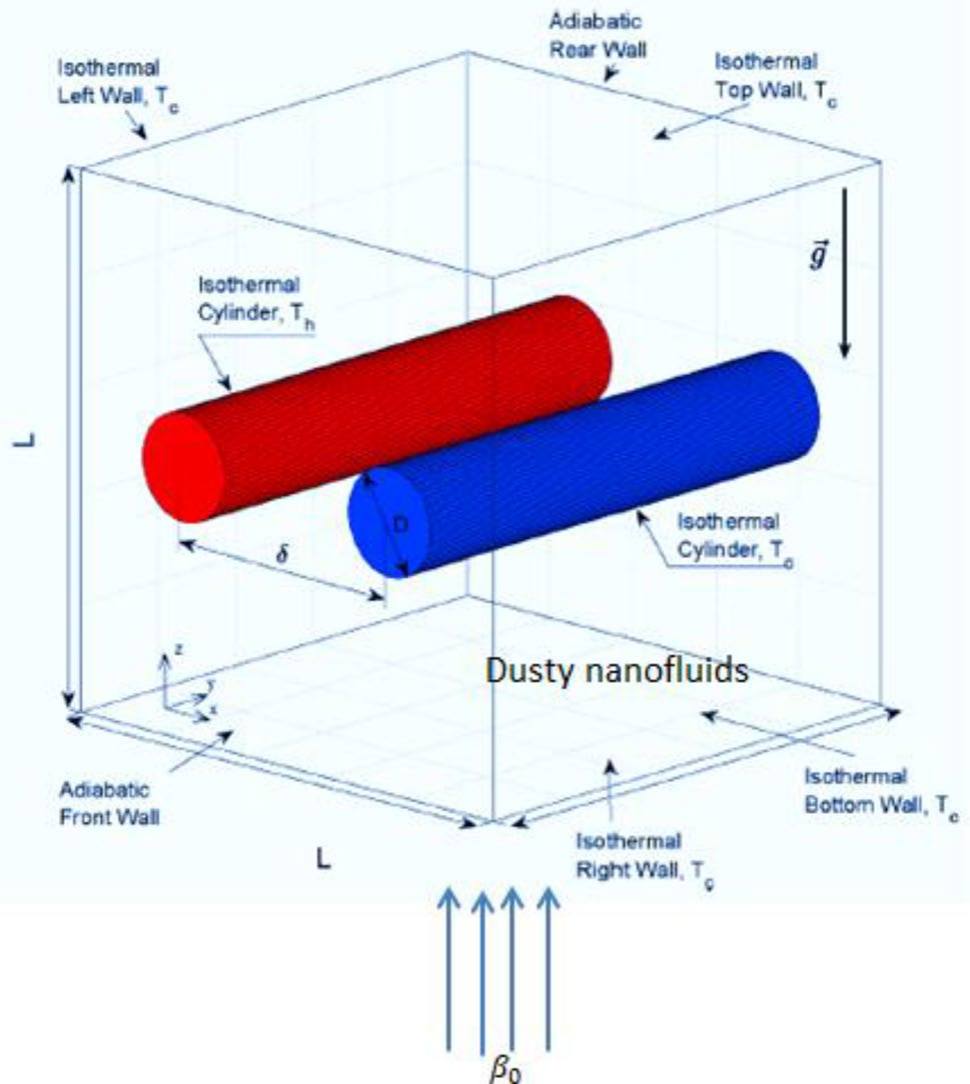
Physical model and problem conditions.

Consider a time-dependent and three dimensional flow within a porous cubic container in the presence of a magnetic field as depicted in Fig. 1. The mixture is a nanofluid contains dusty particles while the domain contains two-cylinders. One of the cylinders is hot ($$T={T}_{h})$$ and the other is cold ($$T={T}_{c})$$ where ($${T}_{h}\gg {T}_{c})$$; those are separated by a distance $$\delta $$. It is assumed, also, that the porous medium is non-homogeneous in all directions where the permeability and thermal conductivity are varied exponentially in $$x{\text{-}}, y$$- and $$z$$-directions. Additionally, the magnetic field is taken in z-direction with constant strength $${\beta }_{0}$$. Table 1 presents the thermophysical properties of the host fluid and nanoparticles. Impacts of the Brownian motion and thermophores are considered in simulating the nanofluids behaviors. Furthermore, the non-homogeneous nanofluid model is applied to represent this physical case and two systems of equations are introduced as, see^13,24,52^:

**Table 1:** Values of the thermophysical properties, see Corcione et al.^52^.

	Material	$\rho$	$\beta \times {10}^{-5}$	${C}_{P}$	$k$	$\sigma$
Al_2_O_3_	Alumina	3970	0.85	765	40	${\mathbf{1}}\times {{\mathbf{10}}}^{-{\mathbf{10}}}$
Host fluid	${\mathrm{Water }}\,[ {25}\,^{\circ }{\mathrm{C} }]$	997.1	21	4179	0.613	**0.05**
Porous matrix	Glass balls	2700		840	1.05	

Significant values are in [bold].

**Nanofluid phase:**

1 $$\frac{\partial u}{\partial x}+\frac{\partial v}{\partial y }+\frac{\partial w}{\partial z}=0$$

2 $$\begin{aligned}{\rho }_{nf }\left[\frac{1}{\varepsilon }\frac{\partial u}{\partial t}+\frac{u}{{\varepsilon }^{2}}\frac{\partial u}{\partial x}+\frac{v}{{\varepsilon }^{2}}\frac{\partial u}{\partial y}+\frac{w}{{\varepsilon }^{2}}\frac{\partial u}{\partial z}\right]&=-\frac{\partial p}{\partial x}+\frac{{\mu }_{nf}}{\varepsilon }\left(\frac{{\partial }^{2}u}{\partial {x}^{2}}+\frac{{\partial }^{2}u}{\partial {y}^{2}}+\frac{{\partial }^{2}u}{\partial z}\right)-\frac{{\mu }_{nf}}{K\left(x,y,z\right)}u\\ &\quad-\frac{{C}_{F}{\rho }_{nf}}{\sqrt{K\left(x,y,z\right)}}\sqrt{{u}^{2}+{v}^{2}+{w}^{2}} u-{\sigma }_{nf}{B}_{0}^{2}\frac{u}{\varepsilon }+\frac{{\rho }_{p}}{{\tau }_{v}} ({u}_{p}-u)\end{aligned}$$

3 $$\begin{aligned}{\rho }_{nf }\left[\frac{1}{\varepsilon }\frac{\partial v}{\partial t}+\frac{u}{{\varepsilon }^{2}}\frac{\partial v}{\partial x}+\frac{v}{{\varepsilon }^{2}}\frac{\partial v}{\partial y}+\frac{w}{{\varepsilon }^{2}}\frac{\partial v}{\partial z}\right]&=-\frac{\partial p}{\partial y}+\frac{{\mu }_{nf}}{\varepsilon }\left(\frac{{\partial }^{2}v}{\partial {x}^{2}}+\frac{{\partial }^{2}v}{\partial {y}^{2}}+\frac{{\partial }^{2}v}{\partial z}\right)-\frac{{\mu }_{nf}}{K\left(x,y,z\right)}v\\ &\quad -\frac{{C}_{F}{\rho }_{nf}}{\sqrt{K\left(x,y,z\right)}}\sqrt{{u}^{2}+{v}^{2}+{w}^{2}} v-{\sigma }_{nf}{B}_{0}^{2}\frac{v}{\varepsilon }+\frac{{\rho }_{p}}{{\tau }_{v}} ({v}_{p}-v)\end{aligned}$$

4 $$\begin{aligned}{\rho }_{nf }\left[\frac{1}{\varepsilon }\frac{\partial w}{\partial t}+\frac{u}{{\varepsilon }^{2}}\frac{\partial w}{\partial x}+\frac{v}{{\varepsilon }^{2}}\frac{\partial w}{\partial y}+\frac{w}{{\varepsilon }^{2}}\frac{\partial w}{\partial z}\right]&=-\frac{\partial p}{\partial z}+\frac{{\mu }_{nf}}{\varepsilon }\left(\frac{{\partial }^{2}w}{\partial {x}^{2}}+\frac{{\partial }^{2}w}{\partial {y}^{2}}+\frac{{\partial }^{2}w}{\partial z}\right)-\frac{{\mu }_{nf}}{K\left(x,y,z\right)}w\\ &\quad -\frac{{C}_{F}{\rho }_{nf}}{\sqrt{K\left(x,y,z\right)}}\sqrt{{u}^{2}+{v}^{2}+{w}^{2}} w+{\left(\rho \beta \right)}_{nf}g\left(T-{T}_{c}\right)+\frac{{\rho }_{p}}{{\tau }_{v}} ({w}_{p}-w)\end{aligned}$$

5 $$\begin{aligned}&{\left[\left(1-\varepsilon \right){\left(\rho {c}_{p}\right)}_{p}+\varepsilon {\left(\rho {c}_{p}\right)}_{nf}\right]\frac{\partial T}{\partial t}+\left(\rho {C}_{p}\right)}_{nf}\left(u\frac{\partial T}{\partial x}+v\frac{\partial T}{\partial y}+w\frac{\partial T}{\partial z}\right)\\ & \quad =\frac{\partial }{\partial x}\left({k}_{eff}(x,y,z)\frac{\partial T}{\partial x}\right)\frac{\partial }{\partial y}\left({k}_{eff}(x,y,z)\frac{\partial T}{y}\right)+\frac{\partial }{\partial z}\left({k}_{eff}(x,y,z)\frac{\partial T}{\partial z}\right)\\ &\qquad +\varepsilon {\left({\rho C}_{p}\right)}_{p}\left[{D}_{B}\left(\frac{\partial \phi }{\partial x} \frac{\partial T}{\partial x}+\frac{\partial \phi }{\partial y} \frac{\partial T}{\partial y}+\frac{\partial \phi }{\partial z} \frac{\partial T}{\partial z}\right)+\frac{{D}_{T}}{T}\left({\left(\frac{\partial T}{\partial X}\right)}^{2}+{\left(\frac{\partial T}{\partial y}\right)}^{2}+{\left(\frac{\partial T}{\partial z}\right)}^{2}\right)\right]+\frac{{\rho }_{p}{c}_{s}}{{\tau }_{T}}\left({T}_{p}-T\right),\end{aligned}$$

6 $$\begin{aligned}\left(\frac{\partial \varphi }{\partial t}+\frac{u}{\varepsilon }\frac{\partial \varphi }{\partial x}+\frac{v}{\varepsilon }\frac{\partial \varphi }{\partial y}+\frac{w}{\varepsilon }\frac{\partial \varphi }{\partial z}\right)&=\frac{\partial }{\partial x}\left({D}_{B}\frac{\partial \varphi }{\partial x}\right)+\frac{\partial }{\partial y}\left({D}_{B}\frac{\partial \varphi }{\partial y}\right)+\frac{\partial }{\partial z}\left({D}_{B}\frac{\partial \varphi }{\partial z}\right)\\ &\quad +\frac{\partial }{\partial x}\left(\frac{{D}_{T}}{T}\frac{\partial T}{\partial x}\right)+\frac{\partial }{\partial y}\left(\frac{{D}_{T}}{T}\frac{\partial T}{\partial y}\right)+\frac{\partial }{\partial z}\left(\frac{{D}_{T}}{T}\frac{\partial T}{\partial z}\right),\end{aligned}$$

**Dusty phase**

7 $$\frac{\partial {u}_{p}}{\partial x}+\frac{\partial {v}_{p}}{\partial y }+\frac{\partial {w}_{p}}{\partial z}=0$$

8 $${\rho }_{p }\left[\frac{\partial {U}_{p}}{\partial \tau }+{U}_{p}\frac{\partial {U}_{p}}{\partial X}+{V}_{p}\frac{\partial {U}_{p}}{\partial Y}+{W}_{p}\frac{\partial {U}_{p}}{\partial Z}\right]=-\frac{\partial {p}_{p}}{\partial x}-\frac{{\rho }_{p}}{{\tau }_{v}} ({u}_{p}-u)$$

9 $${\rho }_{p }\left[\frac{\partial {v}_{p}}{\partial t}+{u}_{p}\frac{\partial {v}_{p}}{\partial x}+{v}_{p}\frac{\partial {v}_{p}}{\partial y}+{w}_{p}\frac{\partial {v}_{p}}{\partial z}\right]=-\frac{\partial {p}_{p}}{\partial y}-\frac{{\rho }_{p}}{{\tau }_{v}} \left({v}_{p}-v\right)$$

10 $${\rho }_{p }\left[\frac{\partial {w}_{p}}{\partial t}+{u}_{p}\frac{\partial {w}_{p}}{\partial x}+{v}_{p}\frac{\partial {w}_{p}}{\partial y}+{w}_{p}\frac{\partial {w}_{p}}{\partial z}\right]=-\frac{\partial {p}_{p}}{\partial z}-\frac{{\rho }_{p}}{{\tau }_{v}} ({w}_{p}-{w})$$

11 $${\rho }_{p}{c}_{s}\left[\frac{\partial {T}_{p}}{\partial t}+{u}_{p}\frac{\partial {T}_{p}}{\partial x}+{v}_{p}\frac{\partial {T}_{p}}{\partial y}+{w}_{p}\frac{\partial {T}_{p}}{\partial z}\right]=-\frac{{\rho }_{p}{c}_{s}}{{\tau }_{T}}({T}_{p}-T)$$

Also, the heterogeneity of the porous medium is represented as:

12 $$K\left(x,y,z\right)={K}_{0} {e}^{{\eta }_{1}x+{\eta }_{2}y+{\eta }_{3}z}$$

13 $${k}_{eff}\left(x,y,z\right)=\left(1-\varepsilon \right){k}_{p}\left(x,y,z\right)+\varepsilon {k}_{nf}=\left(1-\varepsilon \right){k}_{0} {e}^{{\eta }_{1}x+{\eta }_{2}y+{\eta }_{3}z}+\varepsilon {k}_{nf}$$

where the permeability and thermal conductivity of the homogeneous case are ${K}_{0}$ and ${k}_{0}$, respectively and the rates of changing of ${\mathrm{ln}}\,K$ in the three dimensional are ${\upeta }_{1},{\upeta }_{2} ,{\upeta }_{3}$.

Using the following dimensionless parameters:

14 $$\begin{aligned} X&=\frac{x}{L}, \,\,Y=\frac{y}{L},\,\, Z=\frac{z}{L} \tau =\frac{t{\nu }_{f}}{{L}^{2}},\,\, U=\frac{uL}{{\nu }_{f}},\,\, V=\frac{vL}{{\nu }_{f}} ,\,\, W=\frac{wL}{{\nu }_{f}} ,\,\,{ \alpha }_{f}=\frac{{k}_{f}}{{\left(\rho {C}_{P}\right)}_{f}},\\  {U}_{P}&=\frac{{u}_{P}L}{{\nu }_{f}},\,\, {V}_{P}=\frac{{v}_{P}L}{{\nu }_{f}} ,\,\, {W}_{P}=\frac{{w}_{p L}}{{\nu }_{f}} ,\,\, P=\frac{p{L}^{2}}{{\rho }_{nf }{{\nu }_{f}}^{2}},\,\,{P}_{P}=\frac{{P}_{P}{L}^{2}}{{\rho }_{nf }{{\nu }_{f}}^{2} },\,\, \theta =\frac{T-{T}_{c}}{{T}_{h}-{T}_{c}},\,\, {\theta }_{p}=\frac{{T}_{p}-{T}_{c}}{{T}_{h}-{T}_{c}} \\ {\varphi }^{*}&=\frac{\varphi }{{\varphi }_{avg}},\,\, { D}_{B}^{*}=\frac{{D}_{B}}{{D}_{Bo}},\,\, { D}_{T}^{*}=\frac{{D}_{T}}{{D}_{To}},\,\, \delta =\frac{{T}_{c}}{{T}_{h}-{T}_{c}}, \end{aligned}$$

Applying Eq. (14), the following dimensionless systems are obtained:

**Nanofluids phase:**

15 $$\frac{\partial U}{\partial X}+\frac{\partial V}{\partial Y }+\frac{\partial W}{\partial Z}=0$$

16 $$\begin{aligned}\frac{{\rho }_{\mathrm{nf}}}{{\rho }_{f}}\left[\frac{1}{\varepsilon }\frac{\partial U}{\partial \tau }+\frac{U}{{\varepsilon }^{2}}\frac{\partial U}{\partial X}+\frac{V}{{\varepsilon }^{2}}\frac{\partial U}{\partial Y}+\frac{W}{{\varepsilon }^{2}}\frac{\partial U}{\partial Z}\right]&=-\frac{{\rho }_{\mathrm{nf}}}{{\rho }_{f}}\frac{\partial P}{\partial X}+\frac{1}{\varepsilon }\frac{{\mu }_{nf}}{{\mu }_{f}}\left(\frac{{\partial }^{2}U}{\partial {X}^{2}}+\frac{{\partial }^{2}U}{\partial {Y}^{2}}+\frac{{\partial }^{2}U}{\partial {Z}^{2}}\right)-\frac{{\mu }_{nf} }{{{\mu }_{f} K}^{*}\left(X,Y,Z\right)Da}U\\ &\quad-\frac{{\rho }_{nf}}{{\rho }_{f}}\frac{{C}_{F }}{\sqrt{Da{ K}^{*}\left(X,Y,Z\right)}}\sqrt{{U}^{2}+{V}^{2}+{W}^{2}} U-\frac{{\sigma }_{nf}}{{\sigma }_{f}}{Ha}^{2} \frac{U}{\varepsilon }+{\alpha }_{d} {D}_{s} ({U}_{p}-U)\end{aligned}$$

17 $$\begin{aligned}\frac{{\rho }_{\mathrm{nf}}}{{\rho }_{f}}\left[\frac{1}{\varepsilon }\frac{\partial V}{\partial \tau }+\frac{U}{{\varepsilon }^{2}}\frac{\partial V}{\partial X}+\frac{V}{{\varepsilon }^{2}}\frac{\partial V}{\partial Y}+\frac{W}{{\varepsilon }^{2}}\frac{\partial V}{\partial Z}\right]&=-\frac{{\rho }_{\mathrm{nf}}}{{\rho }_{f}}\frac{\partial P}{\partial Y}+\frac{1}{\varepsilon }\frac{{\mu }_{nf}}{{\mu }_{f}}\left(\frac{{\partial }^{2}V}{\partial {X}^{2}}+\frac{{\partial }^{2}V}{\partial {Y}^{2}}+\frac{{\partial }^{2}V}{\partial {Z}^{2}}\right)-\frac{{\mu }_{nf} }{{{\mu }_{f} K}^{*}\left(X,Y,Z\right)Da}V\\&\quad -\frac{{\rho }_{nf}}{{\rho }_{f}}\frac{{C}_{F }}{\sqrt{{ Da K}^{*}\left(X,Y,Z\right)}}\sqrt{{U}^{2}+{V}^{2}+{W}^{2}} U-\frac{{\sigma }_{nf}}{{\sigma }_{f}}{Ha}^{2} \frac{V}{\varepsilon }+{\alpha }_{d} {D}_{s} ({V}_{p}-V)\end{aligned}$$

18 $$\begin{aligned}\frac{{\rho }_{\mathrm{nf}}}{{\rho }_{f}}\left[\frac{1}{\varepsilon }\frac{\partial W}{\partial \tau }+\frac{U}{{\varepsilon }^{2}}\frac{\partial W}{\partial X}+\frac{V}{{\varepsilon }^{2}}\frac{\partial W}{\partial Y}+\frac{W}{{\varepsilon }^{2}}\frac{\partial W}{\partial Z}\right]& =-\frac{{\rho }_{\mathrm{nf}}}{{\rho }_{f}}\frac{\partial P}{\partial Z}+\frac{1}{\varepsilon }\frac{{\mu }_{nf}}{{\mu }_{f}}\left(\frac{{\partial }^{2}W}{\partial {X}^{2}}+\frac{{\partial }^{2}W}{\partial {Y}^{2}}+\frac{{\partial }^{2}W}{\partial {Z}^{2}}\right)-\frac{{\mu }_{nf} }{{{\mu }_{f} K}^{*}\left(X,Y,Z\right)Da}W\\ &\quad-\frac{{\rho }_{nf}}{{\rho }_{f}}\frac{{C}_{F }}{\sqrt{Da{ K}^{*}\left(X,Y,Z\right)}}\sqrt{{U}^{2}+{V}^{2}+{W}^{2}} W+\frac{{\left(\rho \beta \right)}_{nf}}{{\left(\rho \beta \right)}_{f}}\frac{Ra}{Pr}\theta +{\alpha }_{d} {D}_{s} ({W}_{p}-W)\end{aligned}$$

19 $$\begin{aligned}\upgamma \frac{\partial \theta }{\partial \tau }+U\frac{\partial \theta }{\partial X}+V\frac{\partial \theta }{\partial Y}+W\frac{\partial \theta }{\partial Z}&=\frac{{\left(\rho {c}_{p}\right)}_{f}}{{\left(\rho {c}_{p}\right)}_{nf}}\frac{1}{Pr}\left[\frac{\partial }{\partial X}\left(\xi \left(X,Y,Z\right)\frac{\partial \theta }{\partial X}\right)+\frac{\partial }{\partial Y}\left(\xi \left(X,Y,Z\right)\frac{\partial \theta }{\partial Y}\right)+\frac{\partial }{\partial Z}\left(\xi \left(X,Y,Z\right)\frac{\partial \theta }{\partial Z}\right)\right]\\&\quad +\frac{\varepsilon {\left(\rho {c}_{p}\right)}_{f}}{{\left(\rho {c}_{p}\right)}_{nf}}\frac{1}{Le Pr}\left[{D}_{B}^{*}\left[\frac{\partial {\varphi }^{*}}{\partial X}\frac{\partial \theta }{\partial X}+\frac{\partial {\varphi }^{*}}{\partial Y}\frac{\partial \theta }{\partial Y}+\frac{\partial {\varphi }^{*}}{\partial Z}\frac{\partial \theta }{\partial Z}\right]+\frac{{D}_{T}^{*}}{{N}_{BT}}\frac{\left[{\left(\frac{\partial \theta }{\partial X}\right)}^{2}+{\left(\frac{\partial \theta }{\partial Y}\right)}^{2}+{\left(\frac{\partial \theta }{\partial Z}\right)}^{2}\right]}{1+\frac{\theta }{\delta }}\right]\\ &\quad +\frac{2}{3Pr}\frac{{\left(\rho {C}_{p}\right)}_{f}}{{\left(\rho {C}_{p}\right)}_{nf}}{D}_{s}{\alpha }_{d}({\theta }_{p}-\theta )\end{aligned}$$

20 $$\begin{aligned} \left[\frac{\partial {\varphi }^{*}}{\partial \tau }+\frac{U}{\varepsilon }\frac{\partial {\varphi }^{*}}{\partial X}+\frac{V}{\varepsilon }\frac{\partial {\varphi }^{*}}{\partial Y}+\frac{W}{\varepsilon }\frac{\partial {\varphi }^{*}}{\partial Z}\right]&=\frac{1}{Sc}\frac{\partial }{\partial X}\left({D}_{B}^{*}\frac{\partial {\varphi }^{*}}{\partial X}\right)+\frac{1}{Sc}\frac{\partial }{\partial Y}\left({D}_{B}^{*}\frac{\partial {\varphi }^{*}}{\partial Y}\right)+\frac{1}{Sc}\frac{\partial }{\partial Z}\left({D}_{B}^{*}\frac{\partial {\varphi }^{*}}{\partial Z}\right)+\frac{1}{Sc}\frac{\partial }{\partial X}\left(\frac{{D}_{T}^{*}}{{N}_{BT}\left(1+\frac{\theta }{\delta }\right)}\frac{\partial \theta }{\partial X}\right)\\ &\quad +\frac{1}{Sc}\frac{\partial }{\partial Y}\left(\frac{{D}_{T}^{*}}{{N}_{BT}\left(1+\frac{\theta }{\delta }\right)}\frac{\partial \theta }{\partial Y}\right)+\frac{1}{Sc}\frac{\partial }{\partial Z}\left(\frac{{D}_{T}^{*}}{{N}_{BT}\left(1+\frac{\theta }{\delta }\right)}\frac{\partial \theta }{\partial Z}\right)\end{aligned}$$

**Dusty phase:**

21 $$\frac{\partial U{ }_{p}}{\partial X}+\frac{\partial {V}_{p}}{\partial Y }+\frac{\partial {W}_{p}}{\partial Z}=0$$

22 $$\left[\frac{\partial {u}_{p}}{\partial t}+{u}_{p}\frac{\partial {u}_{p}}{\partial x}+{v}_{p}\frac{\partial {u}_{p}}{\partial y}+{w}_{p}\frac{\partial {u}_{p}}{\partial z}\right]=-\frac{{\rho }_{nf }}{{\rho }_{p }}\frac{\partial {P}_{p}}{\partial X}-{\alpha }_{d}({U}_{p}-U)$$

23 $$\left[\frac{\partial {V}_{p}}{\partial \tau }+{U}_{p}\frac{\partial {V}_{p}}{\partial X}+{V}_{p}\frac{\partial {V}_{p}}{\partial Y}+{W}_{p}\frac{\partial {V}_{p}}{\partial Z}\right]=-\frac{{\rho }_{nf }}{{\rho }_{p }}\frac{\partial {P}_{p}}{\partial Y}-{\alpha }_{d} \left({V}_{p}-V\right)$$

24 $$\left[\frac{\partial {W}_{p}}{\partial \tau }+{U}_{p}\frac{\partial {W}_{p}}{\partial X}+{V}_{p}\frac{\partial {W}_{p}}{\partial Y}+{W}_{p}\frac{\partial {W}_{p}}{\partial Z}\right]=-\frac{{\rho }_{nf }}{{\rho }_{p }}\frac{\partial {P}_{p}}{\partial Z}-{\alpha }_{d}({W}_{p}-{W})$$

25 $$\frac{\partial {\theta }_{p}}{\partial \tau }+{U}_{p}\frac{\partial {\theta }_{p}}{\partial X}+{X}_{p}\frac{\partial {\theta }_{p}}{\partial Y}+W\frac{\partial {\theta }_{p}}{\partial Z}=-\frac{2}{3}\frac{{\alpha }_{d}}{\upomega Pr}({\theta }_{p}-\theta )$$

26 $${K}^{*}\left(X,Y,Z\right)=\frac{K(x,y,z)}{{K}_{0}}={e}^{{\eta }_{1}LX+{\eta }_{2}LY+{\eta }_{3}LZ}$$

27 $$\xi \left(X,Y,Z\right)=\left(1-\varepsilon \right){e}^{{\eta }_{1}LX+{\eta }_{2}LY+{\eta }_{3}LZ}+\varepsilon \frac{{k}_{nf}}{{k}_{0}}$$

28 $$\upgamma =\frac{\left(1-\varepsilon \right){\left(\rho {c}_{p}\right)}_{p}+\varepsilon {\left(\rho {c}_{p}\right)}_{nf}}{{\left(\rho {c}_{p}\right)}_{nf}}$$

where

29 $$\begin{aligned} Ra&=\frac{g{\beta }_{f}\left({T}_{h}-{T}_{c}\right){L}^{3}}{{\alpha }_{f}{\nu }_{f}},\,\, Pr=\frac{{\nu }_{f}}{{\alpha }_{f}},\,\, {Ha}^{2}=\frac{{{\sigma }_{f}L}^{2}{B}_{0}^{2}}{{\mu }_{f}},\,\,{\alpha }_{f}=\frac{{k}_{0}}{{\left(\rho {c}_{p}\right)}_{f}},\,\, Da=\frac{{K}_{0}}{{L}^{2} },\,\,Sc=\frac{{\nu }_{\mathrm{f}}}{{D}_{B0}} ,\,\, Le=\frac{{k}_{o}}{{\left(\rho {C}_{P}\right)}_{p} {\varphi }_{avg} {D}_{B0}} , \\ {N}_{BT}&=\frac{{\varphi }_{avg}{D}_{B0}{T}_{c}}{{D}_{T0}\left({T}_{h}-{T}_{c}\right)} ,\,\, { D}_{To}={\varvec{\gamma}}\frac{{\mu }_{f}}{{\rho }_{f}}{\varphi }_{avg} ,\,\, { D}_{B0}=\frac{{K}_{B}{T}_{c}}{3\pi {\mu }_{f}{d}_{p}},\,\,{D}_{s}=\frac{{\rho }_{p} }{{\rho }_{f}} ,\,\,{\alpha }_{d}=\frac{{L}^{2}}{{\nu }_{f}{ \tau }_{\nu }},\,\, \omega =\frac{{c}_{s}}{{c}_{p}},\,\, { \tau }_{t}=\frac{3}{2}{\tau }_{v}\omega \,{Pr} \end{aligned}$$

The boundary conditions can be written as:

30 $$\begin{aligned} &X=1:U=V=W={U}_{p}={V}_{p}={W}_{p}=0,\,\, \theta ={\theta }_{p}=0,\,\,\nabla {\varphi }^{*}.n=-\frac{{D}_{T}^{*}}{{D}_{B}^{*}}\frac{1}{ {\left(1+\frac{\theta }{\delta }\right) N}_{BT}}\nabla \theta .n\\ &Y=0:=V=W={U}_{p}={V}_{p}={W}_{p}=0,\,\, \frac{\partial \theta }{\partial Y}=\frac{\partial {\theta }_{p}}{\partial Y}=0,\,\, \frac{\partial \varphi }{\partial Y}=0,\\ &Y=1:=V=W={U}_{p}={V}_{p}={W}_{p}=0,\,\, \frac{\partial \theta }{\partial Y}=\frac{\partial {\theta }_{p}}{\partial Y}=0,\,\, \frac{\partial \varphi }{\partial Y}=0,\\ &Z=0:=V=W={U}_{p}={V}_{p}={W}_{p}=0,\,\, \theta ={\theta }_{p}=0,\,\,\nabla {\varphi }^{*}.n=-\frac{{D}_{T}^{*}}{{D}_{B}^{*}}\frac{1}{ {\left(1+\frac{\theta }{\delta }\right) N}_{BT}}\nabla \theta .n \\ &Z=1:=V=W={U}_{p}={V}_{p}={W}_{p}=0\,\, \theta ={\theta }_{p}=0,\,\,\nabla {\varphi }^{*}.n=-\frac{{D}_{T}^{*}}{{D}_{B}^{*}}\frac{1}{ {\left(1+\frac{\theta }{\delta }\right) N}_{BT}}\nabla \theta .n\end{aligned}$$

$$\begin{aligned} & {\mathrm{On}}\,\,{\text{the}}\,\, {\text{of}}\,\,{\text{solid}}\,\, {\text{cylinder }}\,\,({\mathrm{hot}}) , \,\,\theta ={\theta }_{p}=1,\,\,\nabla {\varphi }^{*}.n=-\frac{{D}_{T}^{*}}{{D}_{B}^{*}}\frac{1}{ {\left(1+\frac{\theta }{\delta }\right) N}_{BT}}\nabla \theta .n \\ & {\mathrm{On}}\,\,{\text{the}}\,\,{\text{of}}\,\,{\text{solid}}\,\,{\text{cylinder}}\,\,({\mathrm{cold}}),\,\, \theta ={\theta }_{p}=0, \,\,\nabla {\varphi }^{*}.n=-\frac{{D}_{T}^{*}}{{D}_{B}^{*}}\frac{1}{ {\left(1+\frac{\theta }{\delta }\right) N}_{BT}}\nabla \theta .n \end{aligned}$$

The thermophysical properties are given as:

31a $$\frac{{\mu }_{nf}}{{\mu }_{f}}=\frac{1}{{\left(1-\phi \right)}^{2.5}}$$

31b $$\frac{{\rho }_{nf}}{{\rho }_{f}}=\left(1-\phi \right)+\phi \frac{{\rho }_{np}}{{\rho }_{f}}$$

31c $$\frac{{\left(\rho {c}_{p}\right)}_{nf}}{{\left(\rho {c}_{p}\right)}_{f}}=\left(1-\phi \right)+\phi \frac{{\left(\rho {c}_{p}\right)}_{nf}}{{\left(\rho {c}_{p}\right)}_{f}}$$

31d $$\frac{{\left(\rho \beta \right)}_{nf}}{{\left(\rho \beta \right)}_{f}}=\left(1-\phi \right)+\phi \frac{{\left(\rho \beta \right)}_{np}}{{\left(\rho \beta \right)}_{f}}$$

31e $$\frac{{\sigma }_{nf}}{{\sigma }_{f}}=1.+\frac{3\left(\delta -1\right)\phi }{\left(\delta +2\right)-\left(\delta -1\right)\phi }, \delta =\frac{{\sigma }_{nf}}{{\sigma }_{f}},$$

Since the porous medium is heterogeneous, then the local Nu and average $Nu$ are defined as:

32 $${Nu}_{loc}=\frac{\varepsilon {k}_{nf}+(1-\varepsilon ){k}_{0}{e}^{{\eta }_{1}LX+{\eta }_{2}LY+{\eta }_{3}LZ}}{\varepsilon {k}_{f}+(1-\varepsilon ){k}_{0}{e}^{{\eta }_{1}LX+{\eta }_{2}LY+{\eta }_{3}LZ}} {\left.\frac{\partial \theta }{\partial X}\right|}_{X=0}$$

33 $${Nu}_{av}={\int }_{Z=0}^{1}{\int }_{Y=0}^{1}Nu dYdZ$$

## Numerical method and validation

The FVM (finite volume method) with a SIMPLE technique is developed, here, to case of 3D and applied to solve the aforementioned systems of the equation. The details of this methodology are given in Patankar^53^ and Ahmed^54^. The discretization forms of the continuity equations, unsteady, convective, diffusive and source terms are given as:

34 $${\oint }_{S}{\varvec{V}}\cdot {\varvec{n}} dS=0$$

35 $$\begin{aligned} &\frac{{\rho }_{\mathrm{nf}}}{{\rho }_{f}}\frac{1}{\varepsilon }\frac{\partial }{\partial \tau }{\int }_{\Omega }{\varvec{V}} d\Omega +\frac{{\rho }_{nf}}{{\rho }_{f}}\frac{{C}_{F }}{\sqrt{Da{ K}^{*}\left(X,Y,Z\right)}}\sqrt{{U}^{2}+{V}^{2}+{W}^{2}} {\int }_{\Omega }{\varvec{V}} d\Omega +{\alpha }_{d} {D}_{s} {\int }_{\Omega }({{\varvec{V}}}_{p}-{\varvec{V}}) d\Omega \\ &\quad +\frac{{\rho }_{\mathrm{nf}}}{{\rho }_{f}}\frac{1}{{\varepsilon }^{2}}{\oint }_{S}({\varvec{V}}{\varvec{V}})\cdot {\varvec{n}}dS+\frac{{\rho }_{\mathrm{nf}}}{{\rho }_{f}}{\oint }_{S}P\cdot ndS-\frac{Pr}{\varepsilon }{\oint }_{S}\left(\frac{{\mu }_{\mathrm{nf}}}{{\mu }_{f}}\nabla V\right)\cdot ndS+\frac{{\mu }_{eff}Pr }{{{\mu }_{nf} K}^{*}\left(X,Y,Z\right)Da}{\int }_{\Omega }{\varvec{V}} d\Omega\\ &\quad -\chi \frac{{\left(\rho \beta \right)}_{nf}}{{\left(\rho \beta \right)}_{f}} PrRa{\int }_{\Omega }\theta d\Omega =0 \end{aligned}$$

**Figure 2:**
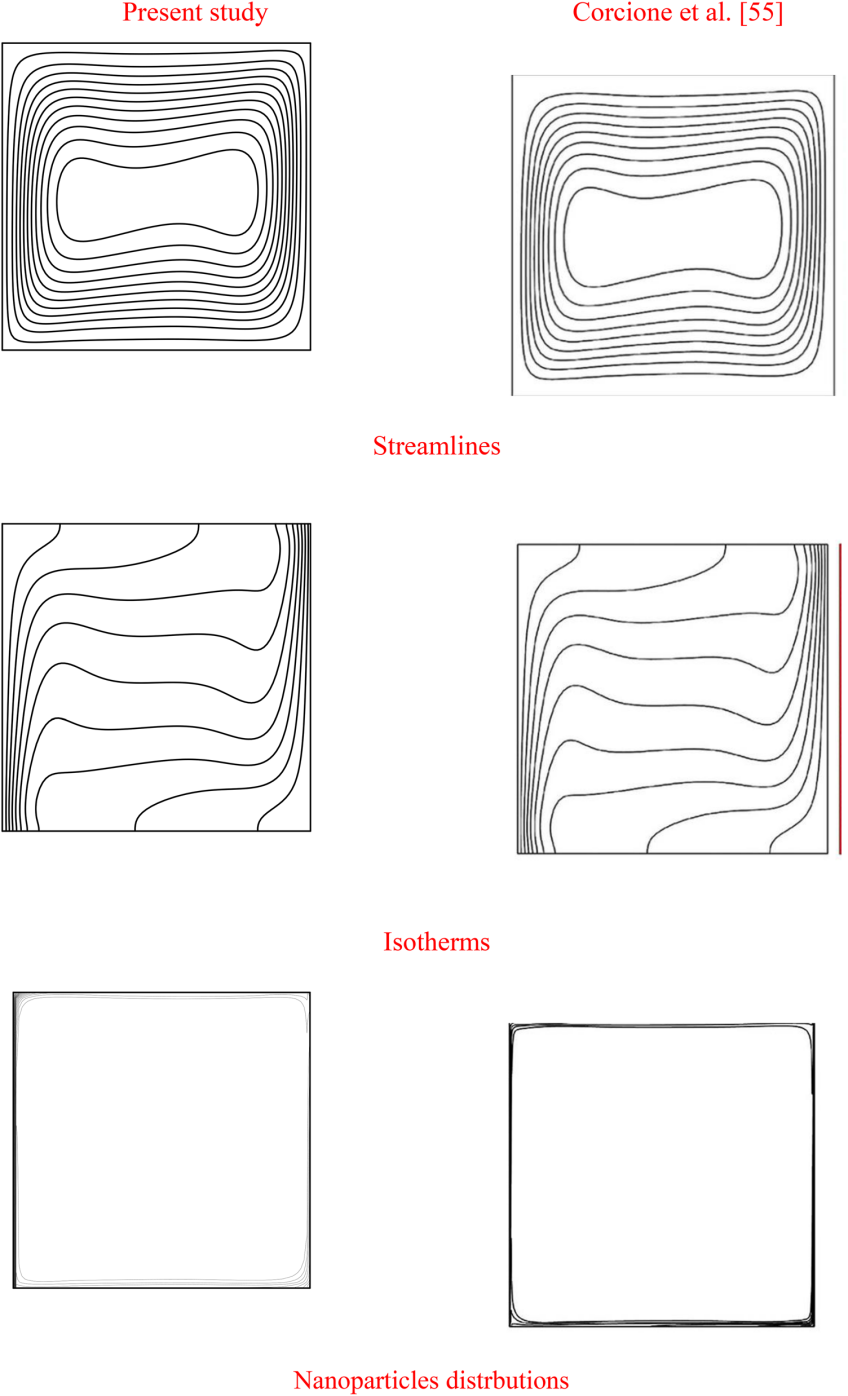
Validation of using the non-homogeneous nanofluids model at $Ra=3.37\times {10}^{5}$ and $\phi =0.04$.

The obtained system is solved iteratively using SUR method with convergence criteria of order ${10}^{-6}$. Table 2 shows the mesh sensitivity at $\delta =0.4,{\eta }_{1}={\eta }_{2}={\eta }_{3}=1.5$, $Da={10}^{-3},Ra={10}^{6}, Ha=10,{\alpha }_{d}=0.1,{D}_{S}=10,{\phi }_{av}=0.02$. It is found the grid size of $41\times 41\times 41$ is enough for all computations. The validation tests of the current study can be divided into two tests. The first one is comparing the results in case of the natural convection due to an inner cylinder within enclosures. This test is presented in Table 3 and an excellent agreement is noted between the results. The 2nd test is comparison of the non-homogeneous model that is presented in Fig. 2. The results revealed that the current outcomes are in very good agreements with the results of Corcione et al.^52^.

**Table 2:** Mesh sensitivity at $\delta =0.4,{\eta }_{1}={\eta }_{2}={\eta }_{3}=1.5$, $Da={10}^{-3},Ra={10}^{6}, Ha=10,{\alpha }_{d}=0.1,{D}_{S}=10,{\phi }_{av}=0.02$.

Grid size	${Nu}_{av}$
$34\times 34\times 34$	7.42488
$41\times 41\times 41$	7.50549
$44\times 44\times 44$	7.66728
$54\times 54\times 54$	8.08446
$64\times 64\times 64$	7.02013

**Table 3:** Comparison of the surface-averaged Nusselt number at the side wall for the different values of $Pr=0.71$.

Ra	Present results	Kim et al.^55^	Difference (%)
${10}^{3}$	1.6847	1.6220	− 3.86
${10}^{4}$	1.6966	1.6905	− 0.36
${10}^{5}$	2.0212	2.0679	2.26

## Results and discussion

In order to understand the physical insight behind this parametric study, a set of graphical illustrations is presented and discussed, here. The distance between the hot/cold cylinders is represented by $\delta$ and its range is taken between 0.3 and 0.6. Also, the range of the Darcy number $Da$, the dusty parameter ${\alpha }_{d}$ and the average nano-parameter ${\phi }_{av}$ are taken, respectively as: ${10}^{-2}\le Da\le {10}^{-5}, 0.001\le {\alpha }_{d}\le 0.1, 0.01\le {\phi }_{av}\le 0.03$. Furthermore, the heterogeneity of the medium properties is taken in $X{-}Y$ plane ${\eta }_{1}={\eta }_{2}=1.5, {\eta }_{3}=0$, in $X{-}Z$ plane ${\eta }_{1}={\eta }_{3}=1.5, {\eta }_{2}=0$ and in $Y-Z$ plane ${\eta }_{2}={\eta }_{3}=1.5, {\eta }_{1}=0$.

**Figure 3:**
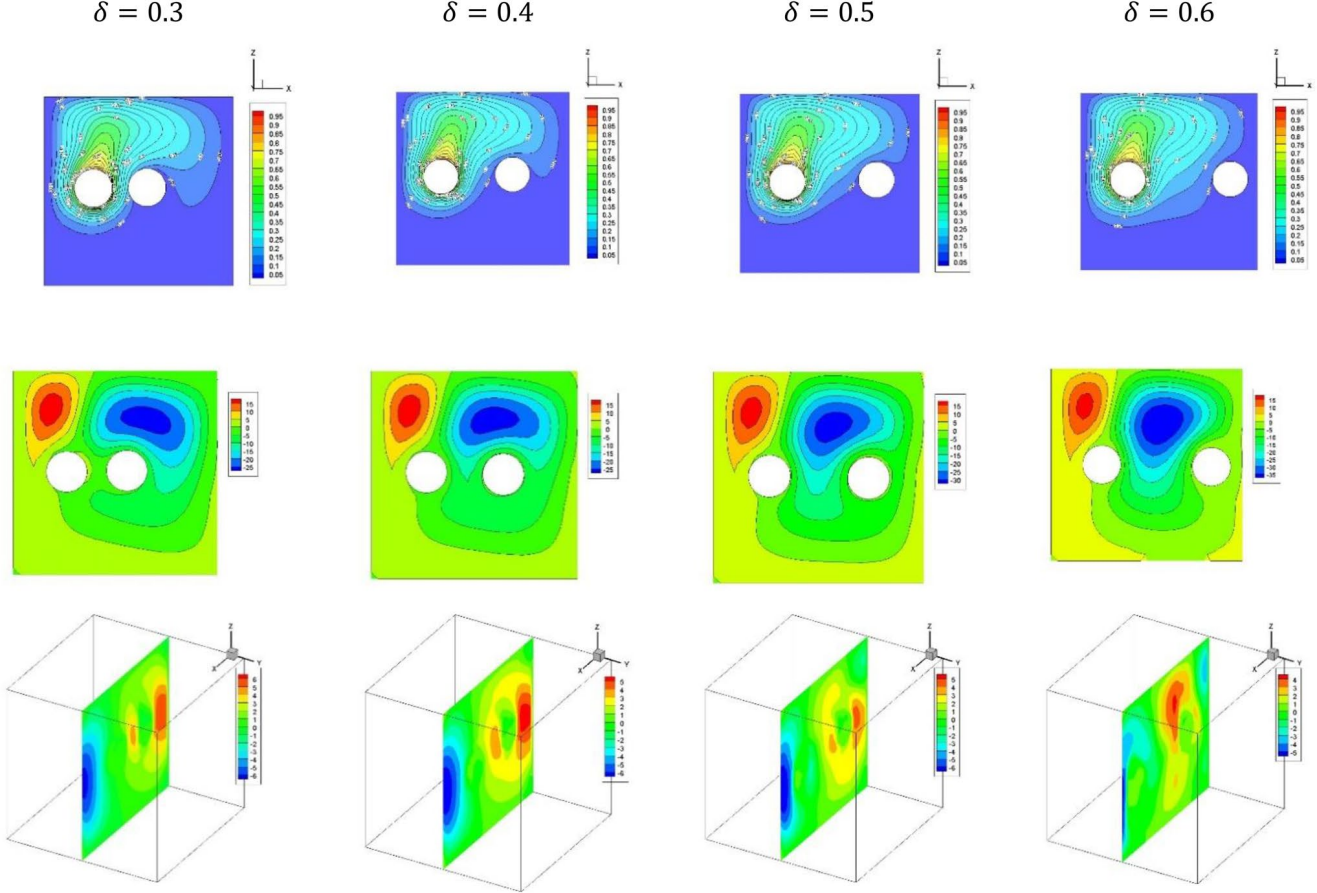
Plots of the temperature distributions, streamlines and dusty velocity ${W}_{p}$ for the variations of the $\delta$ in case of ${(\eta }_{1}={\eta }_{2}={\eta }_{3}=1.5$) at $Da={10}^{-3},Ra={10}^{6}, Ha=10,{\alpha }_{d}=0.1,{D}_{S}=10,{\phi }_{av}=0.02$.

Figure 3 displays plots of the temperature distributions, streamlines and dusty velocity ${W}_{p}$ for the variations of the $\delta$ in case of ${(\eta }_{1}={\eta }_{2}={\eta }_{3}=1.5$) at $Da={10}^{-3},Ra={10}^{6}, Ha=10,{\alpha }_{d}=0.1,{D}_{S}=10,{\phi }_{av}=0.02$. The results revealed that when $\delta$ is increased, the convective transport is enhanced and as results both of the fluid flow and rate of the heat transfer are augmented. The physical explanation of these behaviors is due to the temperature differences within the flow area; those are increased as the cylinders go far from each other.

**Figure 4:**
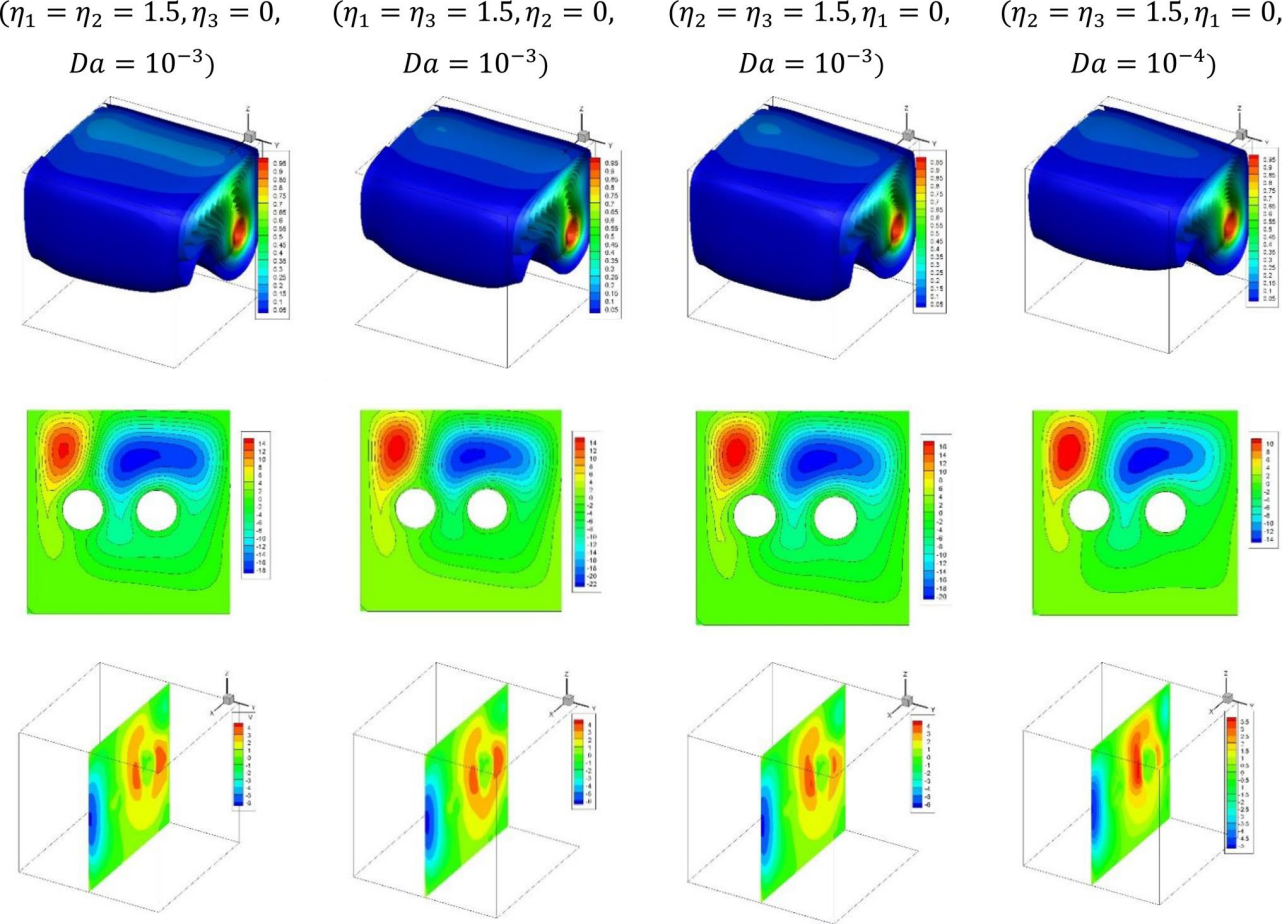
Plots of the temperature distributions, streamlines and dusty velocity ${W}_{p}$ for the variations of (${(\eta }_{1},{\eta }_{2},{\eta }_{3} \,{\mathrm{and}}\, Da)$ at $\delta =0.4, Ra={10}^{6}, Ha=10,{\alpha }_{d}=0.1,{D}_{S}=10,{\phi }_{av}=0.02$.

Figure 4 shows the plots of the temperature distributions, streamlines and dusty velocity ${W}_{p}$ for the variations of (${(\eta }_{1},{\eta }_{2},{\eta }_{3}\,{ \mathrm{and}}\, Da)$ at $Ra={10}^{6}, Ha=10,{\alpha }_{d}=0.1,{D}_{S}=10,{\phi }_{av}=0.02$. Here, the distance between the cylinder is set as 0.4. It is noted that the convective situation is weak when the heterogeneity is considered in the $X{-}Y$ plane ${(\eta }_{1}={\eta }_{2}=1.5, {\eta }_{3}=0, Da={10}^{-3})$ comparing to the other considered cases. Also, the decrease in the Darcy number ${(\eta }_{2}={\eta }_{3}=1.5, {\eta }_{1}=0, Da={10}^{-4})$ causes a reduction in the flow of the nanofluid and dusty velocities due to the decrease in the permeability of the medium.

**Figure 5:**
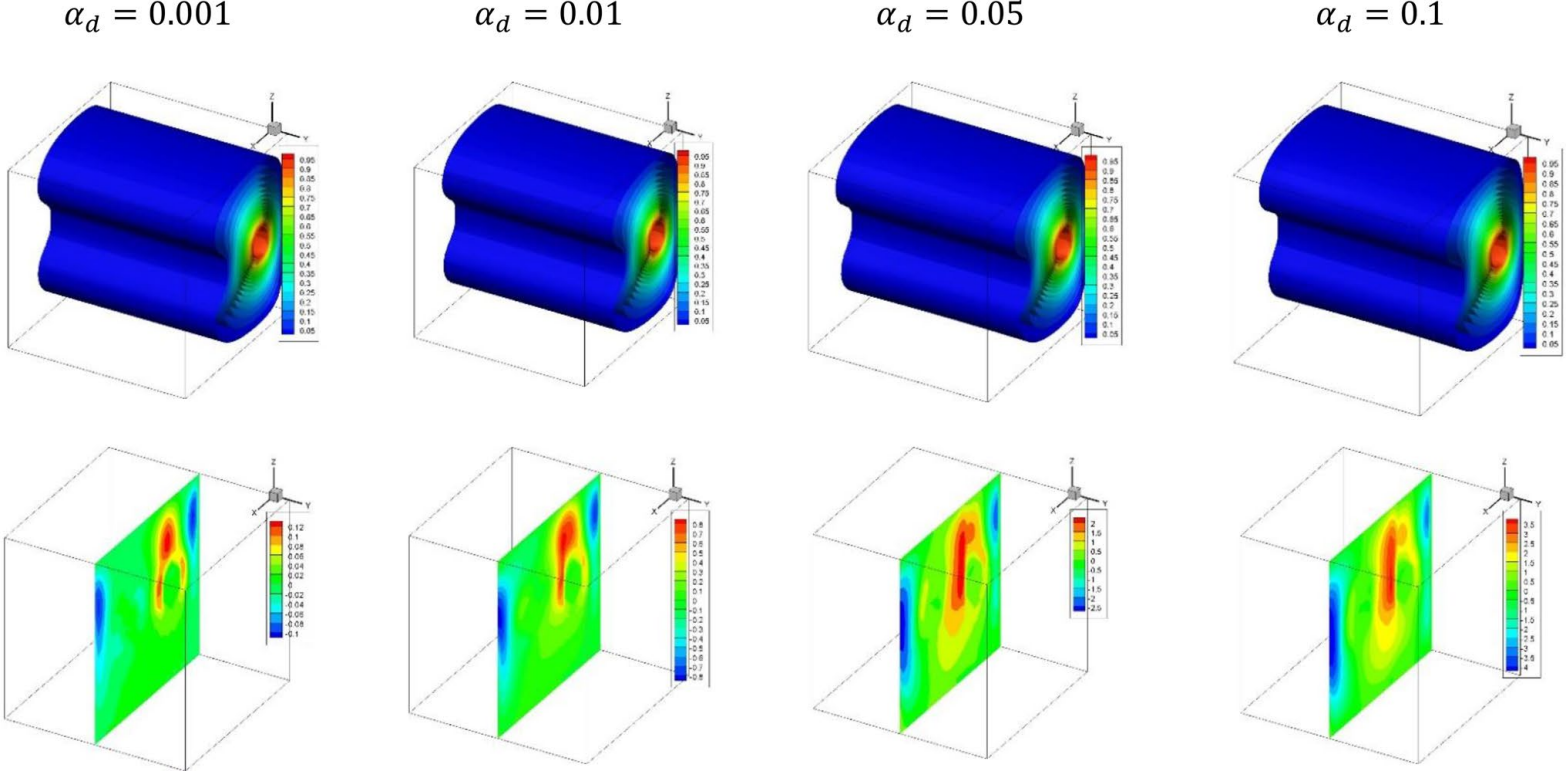
Plots of the dusty temperature distributions and dusty velocity ${W}_{p}$ for the variations of (${\alpha }_{d})$ in case of ${(\eta }_{1}={\eta }_{2}={\eta }_{3}=0$) at $Da={10}^{-3},\delta =0.4, Ra={10}^{6}, Ha=10,{D}_{S}=10,{\phi }_{av}=0.02$.

**Figure 6:**
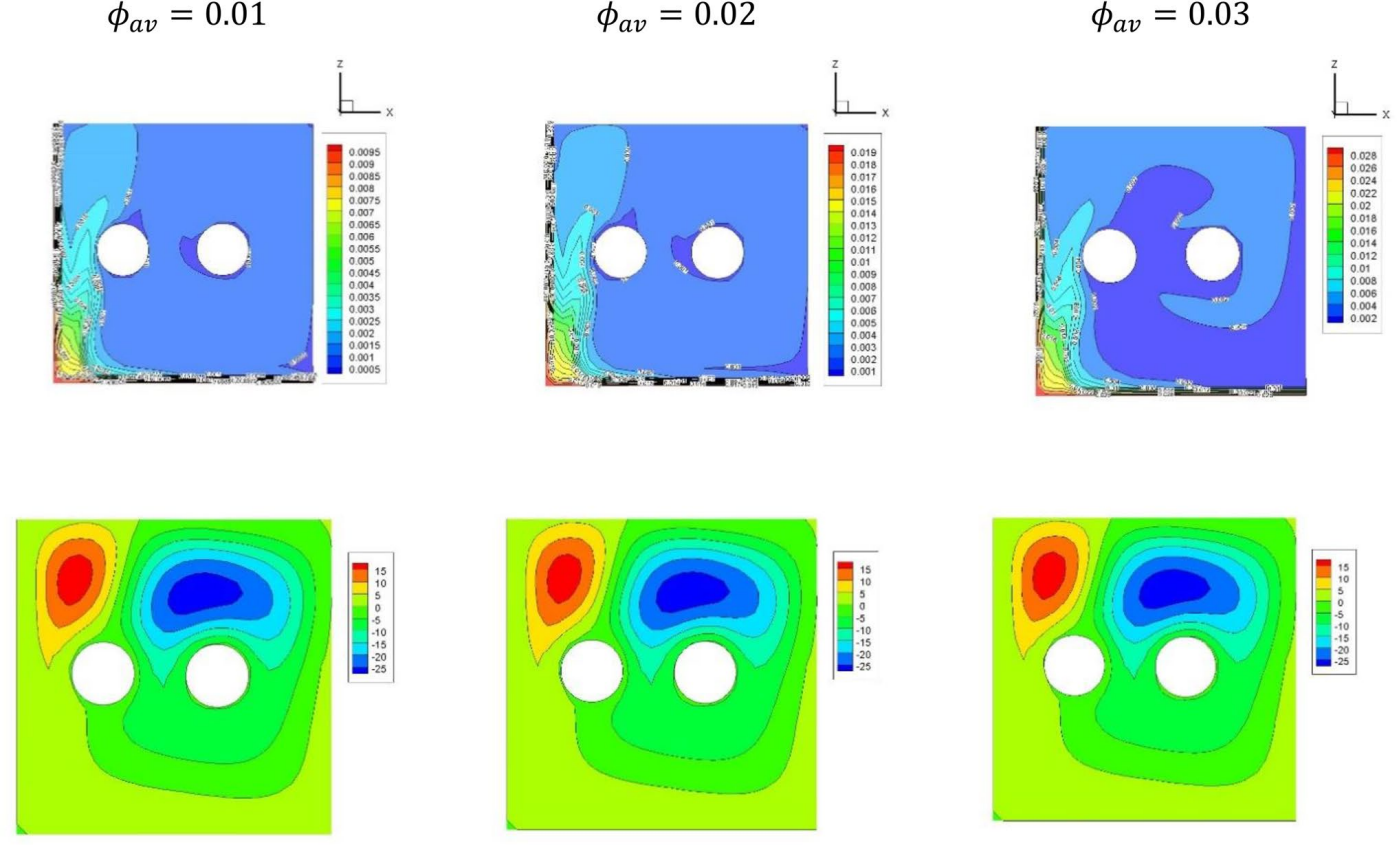
Plots of the nanoparticles distributions and streamlines for the variations of the ${\phi }_{av}$ in case of ${(\eta }_{1}={\eta }_{2}={\eta }_{3}=1.5$) at $Da={10}^{-3},Ra={10}^{6}, \delta =0.4, Ha=10,{\alpha }_{d}=0.1,{D}_{S}=10,\delta =0.4$.

Figure 5 illustrates the plots of the dusty temperature distributions and dusty velocity ${W}_{p}$ for the variations of (${\alpha }_{d})$ in case of ${(\eta }_{1}={\eta }_{2}={\eta }_{3}=0$) at $Da={10}^{-3},\delta =0.4, Ra={10}^{6}, Ha=10,{D}_{S}=10,{\phi }_{av}=0.02$. The outcomes disclosed that the growing in the dusty parameter ${\alpha }_{d}$ enhances the dusty temperature gradients and the dusty velocity ${W}_{p}$. These behaviors returns to the heat exchange between the nanofluid and dusty phases which causes acceleration in the dusty particle velocity. In the same context, Fig. 6 depicts the plots of the nanoparticles distributions and streamlines for the variations of the ${\phi }_{av}$ in case of ${(\eta }_{1}={\eta }_{2}={\eta }_{3}=1.5$) at $Da={10}^{-3},Ra={10}^{6}, \delta =0.4, Ha=10,{\alpha }_{d}=0.1,{D}_{S}=10,\delta =0.4$. The streamlines shows a lack of response for the variation of the average nanoparticle volume fraction while there are a clear augmentation in the both the distribution and maximum values of the nanoparticles volume fraction as ${\phi }_{av}$ is rising.

**Figure 7:**
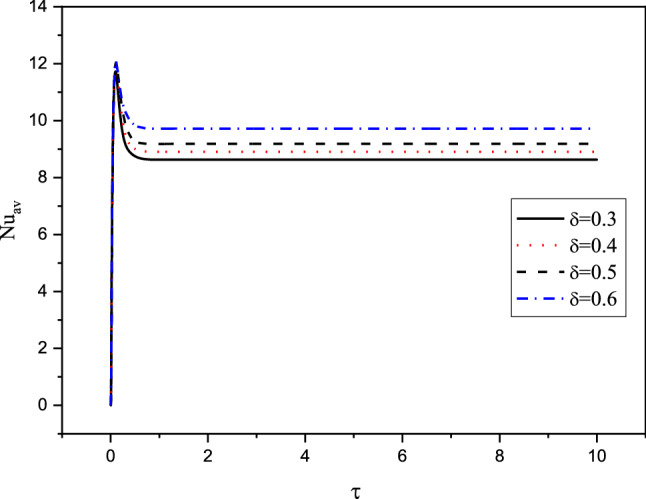
Profiles of the average Nusselt Number for the variations of $\delta$ in case of ${(\eta }_{1}={\eta }_{2}={\eta }_{3}=1.5$) at $Da={10}^{-3},Ra={10}^{6}, Ha=10,{\alpha }_{d}=0.1,{D}_{S}=10,{\phi }_{av}=0.02$.

**Figure 8:**
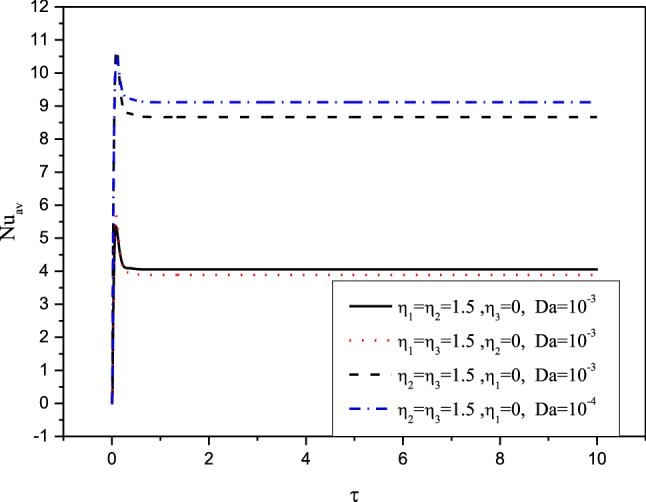
Profiles of the average Nusselt Number for the variations of (${(\eta }_{1},{\eta }_{2},{\eta }_{3}\,{ \mathrm{and}}\, Da)$ at $Ha=10,Ra={10}^{6}, Ha=10,{\alpha }_{d}=0.1,{D}_{S}=10,{\delta =0.4, \phi }_{av}=0.02$.

**Figure 9:**
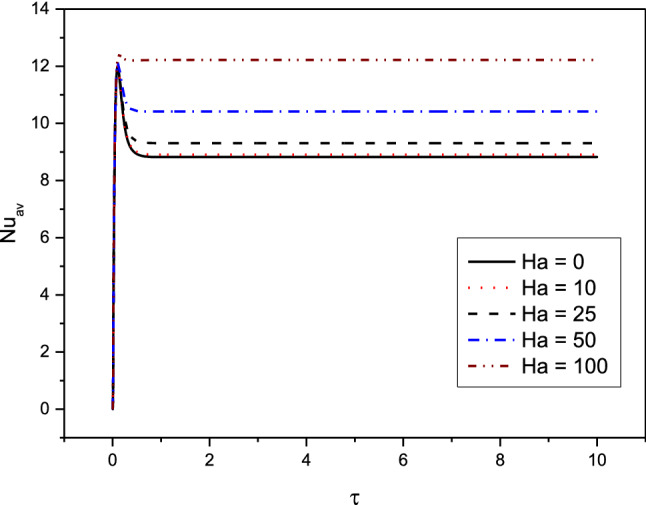
Profiles of the average Nusselt Number for the variations of $Ha$ in case of ${(\eta }_{1}={\eta }_{2}={\eta }_{3}=1.5$) at $Da={10}^{-3},Ra={10}^{6}, \delta =0.4,{\alpha }_{d}=0.1,{D}_{S}=10,{\phi }_{av}=0.02$.

**Figure 10:**
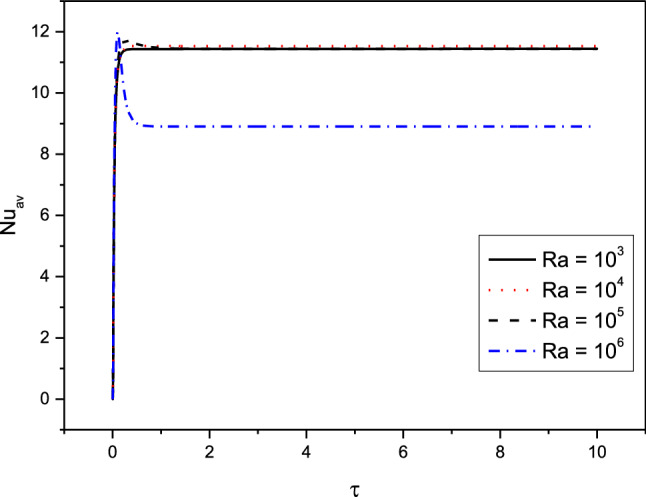
Profiles of the average Nusselt Number for the variations of $Ra$ in case of ${(\eta }_{1}={\eta }_{2}={\eta }_{3}=1.5$) at $Da={10}^{-3},\delta =0.4,{\alpha }_{d}=0.1, Ha=10, {D}_{S}=10,{\phi }_{av}=0.02$.

Figures 7, 8, 9 and 10 displays the profiles of the average Nusselt coefficient ${Nu}_{av}$ for the progressing of the time under impacts of the distance between the cylinders, various cases of the heterogeneity of the medium and several values of the Hartmann number Ha. The results revealed that after a while ($\tau \ge 1.5$), the values of ${Nu}_{av}$ are stable and there are no any perturbations in their profiles. Additionally, clear enhancements are seen in values of ${Nu}_{av}$ as $\delta$ and $Ha$ are rising. The figures, also, disclosed that the cases of the heterogeneity in $X{-}Y$ and $X{-}Z$ directions give the lowest values of ${Nu}_{av}$ due to the decrease in the permeability in these directions.

**Figure 11:**
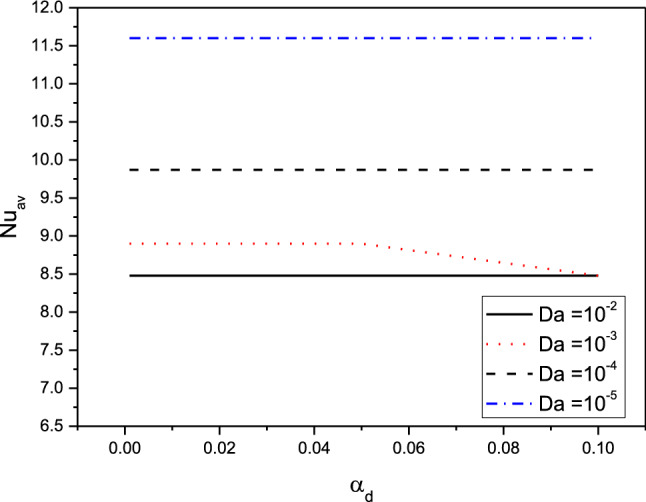
Profiles of the average Nusselt Number for the variations of ${\alpha }_{d} \,and \,Da$ in case of ${(\eta }_{1}={\eta }_{2}={\eta }_{3}=1.5$) at $Ra={10}^{6}, \delta =0.4,Ha=10,{D}_{S}=10,{\phi }_{av}=0.02$.

**Figure 12:**
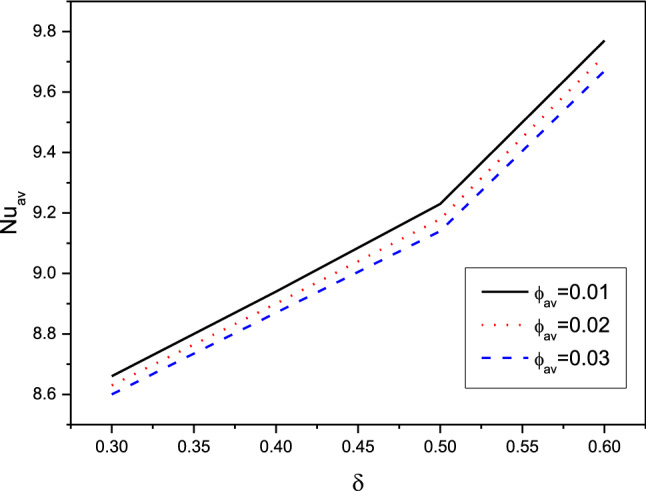
Profiles of the average Nusselt Number for the variations of $\delta \,and\, {\phi }_{av}$ in case of ${(\eta }_{1}={\eta }_{2}={\eta }_{3}=1.5$) at $Ra={10}^{6},Da={10}^{-3},Ha=10,{D}_{S}=10,{\alpha }_{d}=0.1.$

Figures 11 and 12 illustrate the profiles of ${Nu}_{av}$ for the variations of ${\alpha }_{d} , Da, \delta \,{\mathrm{and}}\, {\phi }_{av}$ in case of ${(\eta }_{1}={\eta }_{2}={\eta }_{3}=1.5$) at $Ra={10}^{6}, \delta =0.4,Ha=10,{D}_{S}=10,{\phi }_{av}=0.02$. The dusty coefficient ${\alpha }_{d}$ has no slightly influences on the values of ${Nu}_{av}$ while ${Nu}_{av}$ is augmented, clearly as $Da$ is decreased. Additionally, as stated later, the rising in distance between the cylinders enhances the temperature differences and hence ${Nu}_{av}$ is growing. Furthermore, the increasing values of ${\phi }_{av}$ cause a weakness in the convective situation and hence ${Nu}_{av}$ is reduced.

## Conclusions

Numerical simulations have been carried out for the 3D magnetic convective transport of dusty nanofluid within 3D cubic domain filled by porous material. The nanofluid behavior is presented using the non-homogeneous nanofluid model and the non-Darcy model is applied for the flow through the medium. Two-systems of PDE's are presented for the nanofluid and dusty phases and the magnetic influences are taken in Z-direction. As a new addition in this type of the flow, the heterogeneity of the medium properties, namely, permeability and thermal conductivity are analyzed. The following major findings can be summarized:

- The three dimensional convective transport of dusty nanofluids can be controlled using two inner isothermal cylinders.
- The growing in the distance between the cylinders enhances the three dimensional dusty flow and the rate of the heat transfer.
- The cases of the heterogeneity in $X{-}Y$ and $X{-}Z$ directions have the lowest rate of the heat transfer.
- The rising in Hartmann number enhances the temperature gradients and hence the Nusselt number is augmented.

## List of symbols

${B}_{0}$: Magnetic field strength
$${c}_{F}$$: Inertia coefficient
${c}_{p}$: Specific heat at constant pressure $\left({\mathrm{J}}\, {\mathrm{kg}}^{-1 }\, {\mathrm{K}}^{-1}\right)$
${c}_{s}$: Specific heat for the dust particles $\left({\mathrm{J}}\, {\mathrm{kg}}^{-1 }\, {\mathrm{K}}^{-1}\right)$
$$Da$$: Darcy number
${D}_{B}$: Brownian diffusion coefficient
$${D}_{T}$$: Thermophoresis coefficient
${D}_{s}$: Ratio of the mixture densities
$${\mathrm{g}}$$: Gravity acceleration $$\left({\mathrm{m}} \,{\mathrm{s}}^{-2}\right)$$
$$Ha$$: Hartmann number
$$k$$: Thermal conductivity $$\left({\mathrm{W}}\, {\mathrm{m}}^{-1}\, {\mathrm{K}}^{-1}\right)$$
$K$: Porous medium permeability $({\mathrm{m}}^{2})$
$$L$$: Length (m)
$$L$$e: Lewis number
${N}_{BT}$: Ratio of Brownian to thermophoretic diffusivity
$Nu$: Local Nusselt number
${Nu}_{av}$: Average Nusselt number
$$P$$: Pressure $$\left({\mathrm{N}}\, {\mathrm{m}}^{-2}\right)$$
$$Pr$$: Prandt number
$$Ra$$: Rayleigh number
$$Sc$$: Schmidt number
$$T$$: Temperature $$\left({\mathrm{K}}\right)$$
$$t$$: Time $$\left({\mathrm{s}}\right)$$
$\left(u,v,w\right)$: Velocity components in the *x*, y and *z* direction $\left({\mathrm{m}}\, {\mathrm{s}}^{-1}\right)$
$\left(U,V,W\right)$: Dimensionless velocity components in the *x*, y and *z* direction
$\left(x,y,z\right)$: Dimensional Cartesian coordinates in the *x*, y and *z* direction
$\left(X,Y,Z\right)$: Dimensionless Cartesian coordinates

## Greek symbols

$$\alpha $$: Thermal diffusivity $$\left({\mathrm{m}}^{2}\,{\mathrm{s}}^{-1}\right)$$
${\alpha }_{d}$: Dust parameter depending on the relaxation
$\beta$: Coefficient of thermal expansion $\left({\mathrm{K}}^{-1}\right)$
$\varepsilon$: Porosity of porous medium
$$\uptheta $$: Dimensionless temperature
${\upvarphi }$: Volume fraction nanoparticle
${\varphi }^{*}$: Dimensionless volume fraction nanoparticle
$\phi$: Solid volume fraction
$$\mu $$: Dynamic viscosity $$\left({\mathrm{kg}} \,{\mathrm{m}}^{-1 }\, {\mathrm{s}}^{-1}\right)$$
$$\upnu $$: Kinematic viscosity $$\left({\mathrm{m}}^{2}\,{\mathrm{s}}^{-1}\right)$$
$$\uprho $$: Density $$\left({\mathrm{kg}}\, {\mathrm{m}}^{-3}\right)$$
τ: Dimensionless time
${\tau }_{\nu }$: Momentum relaxation time
${\tau }_{T}$: Thermal relaxation time
$\beta$: Coefficient of thermal expansion $\left({\mathrm{K}}^{-1}\right)$
$\gamma$: Specific heat ratio of the mixture
$$\upsigma $$: Electrical conductivity $$(\Omega \, {\mathrm{m}})$$
${\eta }_{1},{\eta }_{2} ,{\eta }_{3}$: Changing rates of ln (K) in the x, y and z, directions

## Subscripts

$$c$$: Cold
$$h$$: Hot
$$f$$: Fluid
$$p$$: Dusty
$$nf$$: Nanofluid
